# Insights into antibiotic resistomes from gut meta-genome-assembled genomes of the free-range chickens

**DOI:** 10.1016/j.vas.2026.100762

**Published:** 2026-07-22

**Authors:** Dongming Qi, Jinyuan Liu, Xue Bai, Binlong Chen, Fengjiao Qiu, Caiyun Sun, Jianyong An, Anqiang Lai, Xiaoyan Li

**Affiliations:** a Chengdu University, China; b Xichang University , China

**Keywords:** Chicken gut microbiome, Antibiotic resistome, Metagenome-assembled genomes, Regional differences

## Abstract

Antibiotic resistance is a growing global threat, and the chicken gut microbiome is a significant reservoir of antibiotic resistance genes (ARGs). To investigate the presence of ARGs in free-range chickens, which are in closer contact with humans and live in closer proximity to human settlements. In this study, we used metagenomic sequencing to characterize the resistome of the chicken gut. We collected 120 fecal samples from free-range chickens across four Chinese provinces and constructed both metagenome-assembled genomes (MAGs) and comprehensive gene catalogs to investigate microbial community structures and ARG distributions. A total of 2,146 MAGs were reconstructed, encompassing 660 species from 26 phyla, forming a comprehensive genomic catalog of the chicken gut microbiota. We identified 254,316 ARGs representing 159 unique resistance genes across 35 antibiotic classes, with multidrug, tetracycline, glycopeptide, and peptide resistance being most prevalent. Notably, ARG distribution showed strong regional variation, influenced by environmental factors and local farming practices. Mobile genetic elements (MGEs), averaging 33.8 per MAG, were positively correlated with ARG abundance (*R* = 0.77), underscoring their role in facilitating resistance gene dissemination. Specific MAGs—including strains of Escherichia coli and Klebsiella pneumoniae—harbored hundreds of ARGs and virulence factors, highlighting potential high-risk vectors for resistance spread. Our findings reveal a diverse and regionally dynamic antibiotic resistome in the chicken gut, shaped by microbial composition, environment, and host factors. This study provides a valuable genomic resource and emphasizes the need for targeted interventions and surveillance strategies to mitigate antibiotic resistance in poultry production systems.

## 1 Introduction

Antibiotic resistance is one of the most urgent global health challenges (Bai et al., 2024), with projections suggesting it could cause up to 10 million deaths annually by 2050 if current misuse of antibiotics continues unchecked (Pulingam et al., 2022). The gut microbiome—a diverse community of microorganisms inhabiting the gastrointestinal tract (Li et al., 2026; M. Yang et al., 2022)—plays a central role in shaping the antibiotic resistome, the collective reservoir of all ARGs present in an ecosystem (Anthony et al., 2021). The chicken gut microbiome is of particular concern due to its centrality in food production and potential for resistance gene transmission to humans via the food chain (Diaz Carrasco et al., 2019).

Advancements in culture-independent metagenomic approaches have dramatically expanded our understanding of the taxonomic and functional diversity of the chicken gut microbiota (Feng et al., 2021). Like other animal hosts (Bai et al., 2025; Bai et al., 2024), the chicken gut is dominated by four major bacterial phyla: Firmicutes, Bacteroidetes, Actinobacteria, and Proteobacteria (Feng et al., 2021). Although the microbial gene content in chickens shows considerable divergence from that of humans and pigs (Huang et al., 2018), the caeca, the site of highest microbial density, play a vital role in host health through fermentation of complex carbohydrates and production of short-chain fatty acids (Chen et al., 2023). Previous studies have demonstrated that free-range chickens tend to harbor fewer ARGs compared to commercial broilers, likely due to reduced antibiotic exposure and more diverse environmental microbial interactions (Hegde et al., 2016; Liu et al., 2020). Moreover, these free-range chickens have closer contact with humans, which may facilitate the transmission of antimicrobial resistance genes.

The advent of metagenomics, which enables direct analysis of DNA extracted from environmental samples (Gao et al., 2024; T. Wang, P. Li, et al., 2024), has revolutionized microbiome research (Taş et al., 2021) by allowing the reconstruction of MAGs and the development of large-scale gene catalogs (Cansdale & Chong, 2024), including the chicken gut (Huang et al., 2018). Such resources are instrumental in characterizing ARG diversity, abundance, distribution, host range, and potential transmission mechanisms. Notably, the chicken gut microbiome has been increasingly recognized as a reservoir of ARGs (Feng et al., 2021). Studies show that chickens raised under varying farming conditions harbor distinct microbiomes with differing ARG burdens (Hegde et al., 2016; Zhou et al., 2012), suggesting that agricultural practices and environmental factors significantly shape the resistome landscape. Furthermore, recent work has identified specific microbial taxa linked to ARG carriage in chickens, highlighting the importance of understanding these associations for resistance mitigation (Juricova et al., 2021; J. Yang et al., 2022). In many parts of the world, chickens are still raised in a free-range manner. They live very close to humans, but there haven't been many studies on the abundance of their drug-resistant genes and the risk of their spread from the metagenome level.

In this study, we performed a comprehensive analysis of the chicken gut antibiotic resistome by leveraging MAGs and gene catalogs generated from 120 fecal samples collected across four provinces in China. By integrating genomic, taxonomic, and functional data, we aimed to elucidate the genetic underpinnings of resistance, identify potential ARG reservoirs and vectors, and explore the ecological and regional dynamics of ARG distribution. This is the first MAG-based characterization of ARG profiles in free-range chickens from rural China—spanning multiple altitudes and involving chickens with close daily human contact—provides strain-resolved insights into ARG hosts and their potential implications for human health. These findings contribute to a deeper understanding of resistance gene dissemination in agricultural systems and support the development of more effective, regionally tailored strategies to manage antibiotic resistance in poultry and safeguard public health.

## 2 Materials and methods

### 2.1 Sample collection and DNA extraction

A total of 120 chicken fecal samples were collected from four provinces in China: Sichuan, Yunnan, Guizhou, and Jiangsu. Detailed metadata, including chicken breed, province, city, county/district, village/farm, altitude, and GPS coordinates, are provided in Supplementary Table S1. Fresh fecal samples were collected non-invasively from free-range chickens without causing any distress or harm to the animals. All sampling sites represent family farming and small-scale free-range domestic poultry systems, raise 50–200 free-range chickens, which is typical for rural household-level production. The chickens are raised under free-range conditions and feed themselves naturally, without industrial management or controlled interventions. All fecal samples were collected directly from the cloaca using sterile swabs to avoid environmental contamination. All samples were placed on dry ice immediately after collection, transported to the laboratory, and stored at −80°C until further processing to preserve microbial DNA integrity. DNA was extracted using the QIAamp Fast DNA Stool Mini Kit (Qiagen, Hilden, Germany), following the manufacturer’s protocol. DNA purity and concentration were assessed using Nanodrop and Qubit assays.

### 2.2 Metagenomic sequencing

Genomic DNA was sheared to an average fragment size of ∼350 bp to construct paired-end libraries. Adapters containing full sequencing primer hybridization sites were ligated to the blunt ends of the fragments. Sequencing was performed using the DNBSEQ-T7 platform (BGI, China) by Beijing Novogene Technology Co., Ltd.

### 2.3 Metagenome bioinformatics analysis

Initial sequence quality control and assembly were performed on the Majorbio Cloud Platform (www.majorbio.com). Adapter trimming and removal of reads <50 bp or with a quality score <20 were conducted using fastp (version 0.23.0) (Chen et al., 2018). Additional filtering, trimming, and quality screening were performed using SeqPrep (https://github.com/jstjohn/SeqPrep) and Sickle (version 1.33, https://github.com/najoshi/sickle). Host contamination was removed by aligning clean reads to the chicken genome (GRCg7b) using BWA (version 0.7.9a) (Li & Durbin, 2009), and mapped reads were discarded. The remaining reads were assembled into contigs using MEGAHIT (version 1.1.2) (Li et al., 2015).

### 2.4 Metagenomic binning and quality control of MAGs

Genome binning was performed using three tools—MetaBAT2 (version 2.12.1) (Kang et al., 2015), MaxBin2 (version 2.2.5) (Alneberg et al., 2014), and CONCOCT (version 0.5.0) (Wu et al., 2016)—with default parameters. DAS Tool (version 1.1.0) (Sieber et al., 2018) was used to integrate results and generate a non-redundant set of genome bins. MAG quality refinement was conducted using RefineM (version 0.0.24) (Parks et al., 2017), which filtered out contigs with anomalous GC content, tetranucleotide signatures, coverage, or conflicting taxonomic assignments. MAG completeness and contamination were assessed with CheckM (version 1.0.12) (Parks et al., 2015) using lineage-specific marker genes. Only MAGs with ≥50% completeness and <10% contamination were retained for further analysis (Parks et al., 2017).

Average nucleotide identity (ANI) among MAGs was computed using Mash (Ondov et al., 2016), and redundant MAGs were dereplicated with dRep (version 3.4.2) (Olm et al., 2017) based on ≥99% ANI. The highest-quality representative MAG from each cluster was selected for downstream analysis. MAG coverage was estimated using CoverM (version 0.6.1) [CoverM GitHub repository]. Taxonomic classification was performed using GTDB-Tk (version 2.3.0) (Parks et al., 2018), based on 120 universal single-copy marker proteins from the Genome Taxonomy Database (GTDB).

### 2.5 Gene prediction and functional annotation

Gene prediction was conducted using Prodigal (version 2.6.3) in metagenomic mode (-p meta) (Hyatt et al., 2010). The resulting gene sequences were functionally annotated against several databases: (1) NR, the NCBI non-redundant protein database, used for broad functional annotation via local BLAST. (2) KEGG, the Kyoto Encyclopedia of Genes and Genomes (accessed in August 2023), which enables systematic linkage of genomic content to high-level functional information. Gene alignment was performed using Diamond (version 5.1; E-value ≤ 1e–5) (Buchfink et al., 2015), followed by annotation with KOBAS 2.0 (Xie et al., 2011). (3) eggNOG database was used to assign COG/KOG/arCOG categories and support phylogenomic annotation. (4) the Carbohydrate-Active enZYmes database was used to annotate enzymes involved in the synthesis and degradation of complex carbohydrates. (5) CARD, the Comprehensive Antibiotic Resistance Database (version 3.0.9) was employed for ARG identification. Built on the Antibiotic Resistance Ontology (ARO), CARD enables classification of resistance genes by target, mechanism, and genetic variant.

ARGs and MGEs were identified from genome annotation data. For each genome, all genes were sorted by their start coordinates. An ARG was considered adjacent to an MGE if either its immediate upstream or downstream gene was annotated as an MGE. The adjacency was further classified into three types based on the position of the neighboring MGE(s): previous gene only, next gene only, or both sides. The overall proportion of ARGs adjacent to MGEs and the genome-wise adjacency frequencies were then calculated.

### 2.6 Gene prediction and functional annotation

All statistical analyses were performed using R (version 4.3.1) unless otherwise specified. Correlation analyses between microbial taxa, environmental variables, ARGs, and MGEs were conducted using Spearman’s rank correlation. To control for false-positive results arising from multiple testing, *P* values were adjusted using the Benjamini–Hochberg false discovery rate (FDR) correction. Associations were considered statistically significant at FDR-adjusted *P* < 0.05. Comparisons of microbial community structure among provinces were assessed using alpha-diversity indices (Shannon, and Simpson) and beta-diversity analysis based on Bray–Curtis distance matrices. Differences in beta diversity were evaluated using PERMANOVA implemented in the vegan package. Differential abundance of MAGs across regions was tested using ANCOM-BC (version 2)(Lin & Peddada, 2020), which accounts for compositional bias and provides bias-corrected estimates of log-fold changes. Only MAGs with prevalence ≥10% across samples were included in these analyses.

## 3 Results

### 3.1 Assembly of 2,146 MAGs from 120 chicken gut microbiome samples

**Fig. 1:**
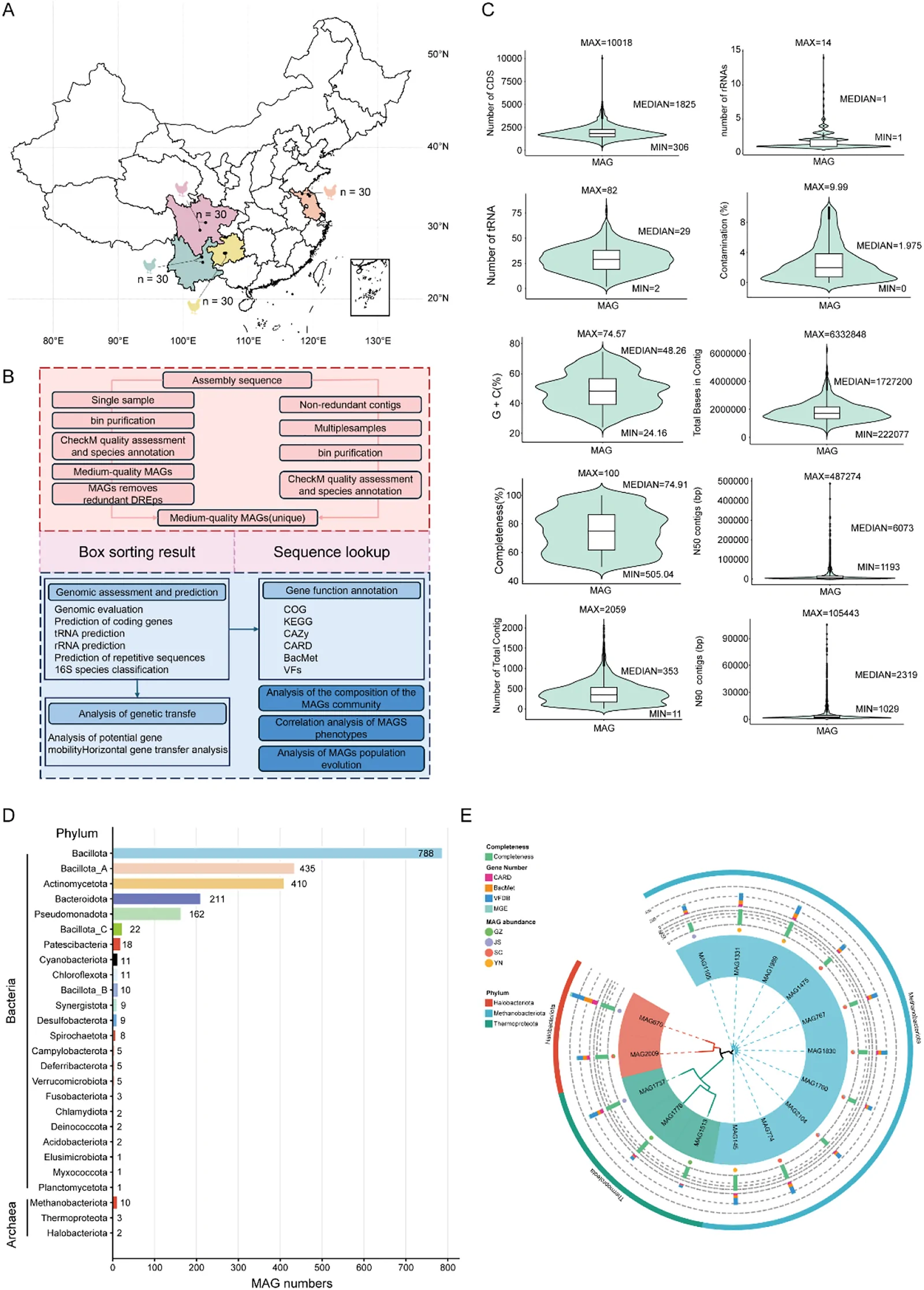
Overview of the metagenome-assembled genome catalog from 120 chicken gut microbiomes. **A** Geographic distribution of sampling sites. **B** Genome quality summary and antibiotic resistance gene (Li et al.) annotation. **C** Assembly statistics including completeness, contamination, CDS count, GC content, N50, and tRNA/rRNA content. Gray dots represent average values. **D** MAG abundance distribution across samples. **E** Circular phylogenetic tree based on conserved marker genes from archaeal MAGs at the phylum level.

A total of 120 chicken fecal samples were collected from four Chinese provinces—Yunnan, Guizhou, Sichuan, and Jiangsu—for metagenome-assembled genome (MAG) reconstruction (Fig. 1A, Supplementary Table S1). Here, we generated 1,371 Gb of raw data, yielding 1,202 Gb of high-quality reads after stringent quality control, with an effective data retention rate of 86.62% (Supplementary Table S2). Following the standards established by the Genomic Standards Consortium (Bowers et al., 2017), deduplication and quality filtering resulted in 2,146 MAGs that met the moderate-quality threshold (≥50% completeness, ≤10% contamination) (Supplementary Table S3). Of these, 865 MAGs were classified as high-quality (>80% completeness, <10% contamination); 223 had >95% completeness and <5% contamination; and five MAGs exhibited >97% completeness and 0% contamination (Table S2, Fig. 1B). 1

Comprehensive genomic metrics were calculated for each MAG, including coding DNA sequence (CDS) counts, rRNA content, contamination rates, G+C content, completeness, total contig length, and N50/N90 statistics (Fig. 1C). Among the assembled MAGs, MAG884 and MAG988 were the most prevalent, each detected in 111 samples (Table S3).

### 3.2 Microbial community composition

The vast majority of reconstructed genomes were bacterial (2,131 MAGs), with only 15 MAGs belonging to archaea. Taxonomic classification using the Genome Taxonomy Database (GTDB) assigned the 2,146 MAGs to 26 phyla, 36 classes, 76 orders, 162 families, 463 genera, and 660 species (Table S2).

**Fig. 2:**
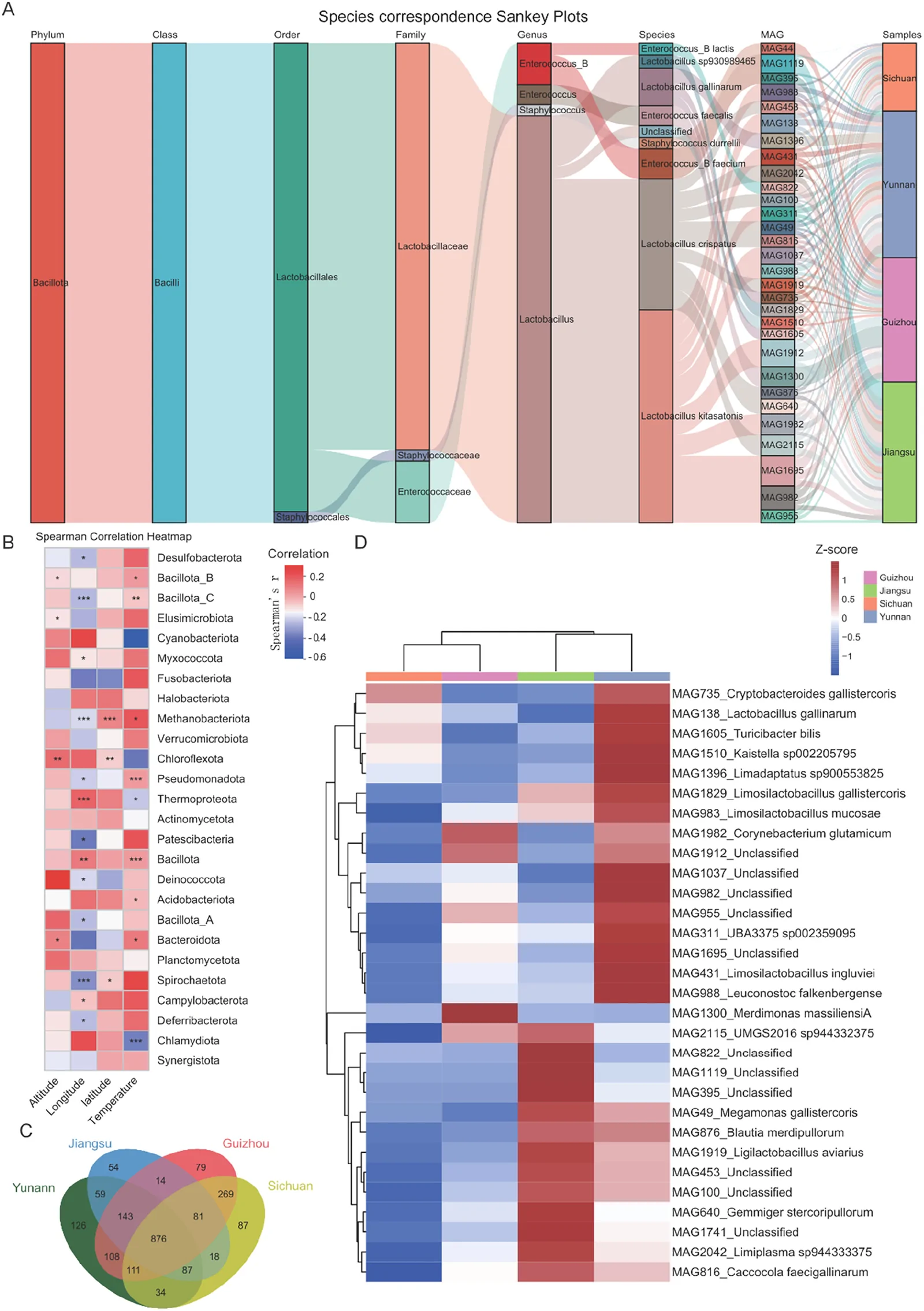
Summary of microbial community composition across 120 chicken gut samples. **A** Sankey diagram showing the taxonomic classification of MAGs and their distribution across the four provinces (SC: Sichuan; GZ: Guizhou; JS: Jiangsu; YN: Yunnan). **B** Heatmap illustrating correlations between environmental variables (altitude, latitude, longitude, temperature) and microbial phyla, statistical significance is indicated as *p* < 0.05 (*), *p* < 0.01 (**), *p* < 0.001 (***). **C** Venn diagram representing shared and unique MAGs among provinces. **D** Heatmap showing enrichment of the top 30 core MAGs across samples.

The chicken gut microbiome was dominated by four major phyla: Bacillota, Bacillota_A, Actinomycetota, and Bacteroidota, which together accounted for over 82.3% of all MAGs. Specifically, Bacillota, Bacillota_A, and Actinomycetota represented 36.87%, 18.28%, and 17.88% of the community, respectively (Fig. 2A, Table S3). Notably, regional differences were evident. For example, Planctomycetota was absent in the Guizhou samples. At the genus level, Lactobacillus (5.42%–9.18%), Limosilactobacillus (5.35%–8.64%), Ligilactobacillus (4.49%–7.33%), and Corynebacterium (2.32%–3.46%) were consistently present across all provinces. Among them, Lactobacillus was most abundant in Jiangsu (9.18%), followed by Zhejiang (7.46%), Yunnan (7.11%), and Sichuan (5.42%). Lactobacillus plays a protective role by producing lactic acid and hydrogen peroxide, which lowers gut pH and inhibits pathogenic bacteria (Su et al., 2021).

To further explore community structure and environmental associations, MAG abundance was visualized using species-level Sankey plots and heatmaps. At the phylum level, the top 30 most abundant MAGs all belonged to Bacillota (Fig. 2A). Deeper taxonomic resolution revealed more complex branching patterns, with several species exhibiting sample-specific enrichment. For example, MAG1300 was significantly enriched in Guizhou samples, potentially due to local environmental conditions (Fig. 2A). Correlation analyses showed that MAG distributions were significantly associated with environmental variables such as altitude, temperature, and geographic coordinates. For instance, Chloroflexota abundance was positively correlated with altitude, whereas Chlamydiota abundance was negatively correlated with temperature (Fig. 2B).

Further comparison of MAGs across provinces revealed 876 core MAGs, including representatives from Bacillota (305), Actinomycetota (165), and Bacteroidota (94). Additionally, 346 MAGs were province-specific: 87 in Sichuan, 126 in Yunnan, 79 in Guizhou, and 87 in Jiangsu (Fig. 2C). A heatmap of the top 30 most abundant shared MAGs showed relatively low abundance in Sichuan but higher levels in Guizhou and Jiangsu (Fig. 2D).

### 3.3 Functional annotation of MAGs

**Fig. 3:**
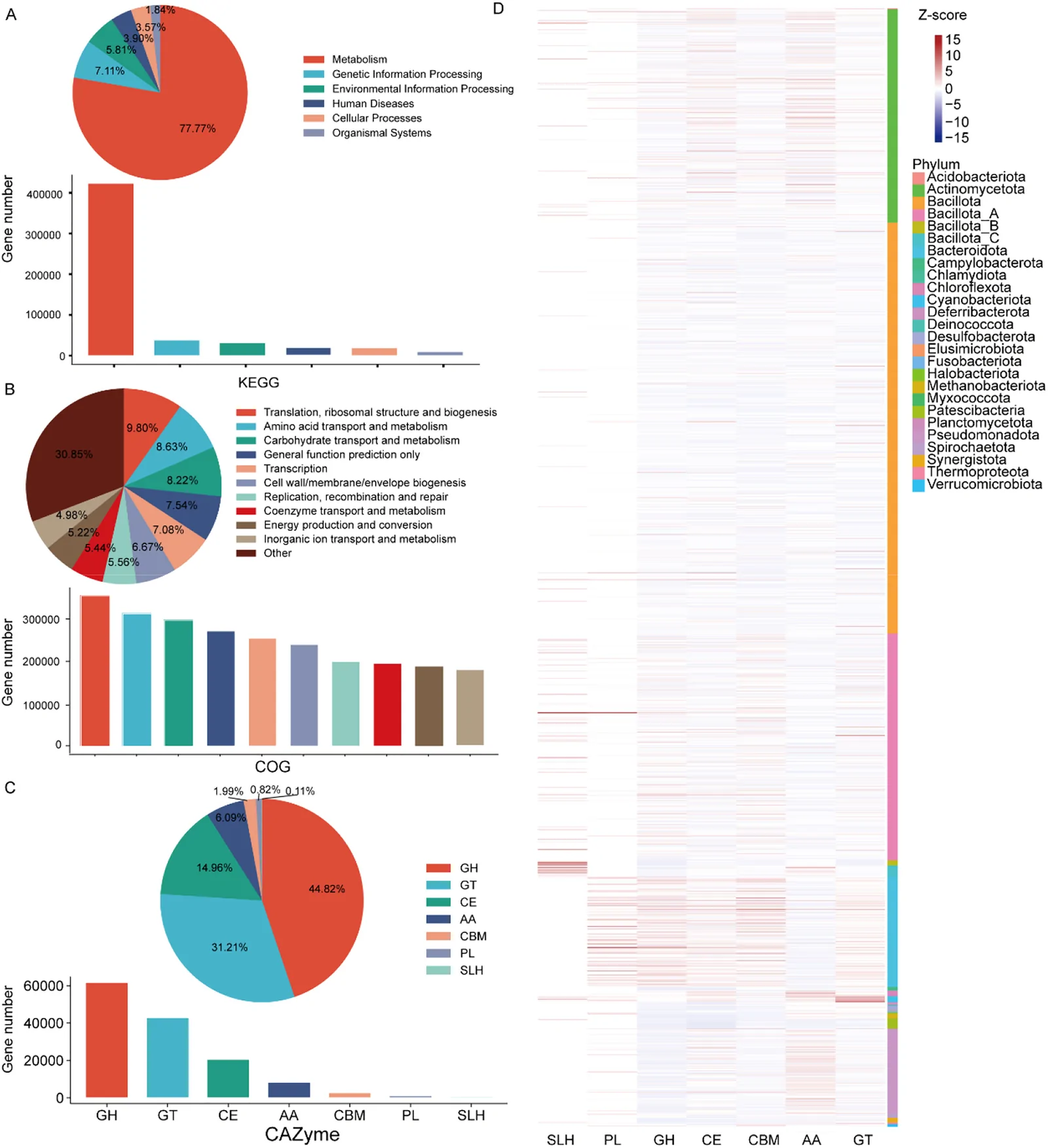
Functional annotation of MAGs from 120 chicken gut samples. **A** KEGG classification of predicted coding genes. **B** COG-based functional classification. **C** CAZyme distribution across MAGs. **D** Heatmap showing correlations between MAGs and CAZy categories at the phylum level.

To explore the functional potential of the chicken gut microbiome, all 2,146 MAGs were annotated against five major databases: NR, COG, KEGG, CAZy, and CARD. KEGG annotations (Fig. 3A) classified predicted proteins into six main metabolic categories, with ∼77.77% assigned to metabolism-related functions. Nearly all MAGs encoded pathways for nucleotide, cofactor, and vitamin metabolism, while only a minority lacked pathways related to the biodegradation of xenobiotics.

COG annotations revealed that only 1.39% and 18.17% of MAGs encoded genes involved in chromatin structure/dynamics and RNA processing/modification, respectively (Fig. 3B). These low proportions suggest that most chicken gut microbes likely regulate gene expression through mechanisms other than chromatin remodeling, possibly reflecting stable genome architecture and low reliance on structural plasticity for environmental adaptation.

Annotation against the CAZy database identified 137,607 carbohydrate-active enzyme (CAZyme) genes, classified into seven major CAZy families (Supplementary Table S4). Glycoside hydrolases (GHs) and glycosyltransferases (Pal et al.) were the most abundant, followed by carbohydrate esterases (CEs) and auxiliary activities (AAs) (Fig. 3C). Correlation analysis revealed phylum-level associations between MAGs and specific CAZy categories, indicating that functional carbohydrate metabolism is phylum-specific (Fig. 3D).

BacMet predictions identified MAG2050 (Klebsiella pneumoniae), MAG1267 (Janthinobacterium sp001758725), and MAG1655 (Leclercia adecarboxylata_C) as harboring the highest numbers of BacMet-associated genes (638, 562, and 536, respectively). Similarly, virulence gene prediction using virulence factor database (VFDB) showed that MAG1267, MAG322 (Escherichia coli), and MAG1655 contained the highest counts of virulence factors (934, 832, and 815, respectively), indicating potential risks associated with pathogenicity and environmental survival.

### 3.4 Antibiotic resistance genes profiles

**Fig. 4:**
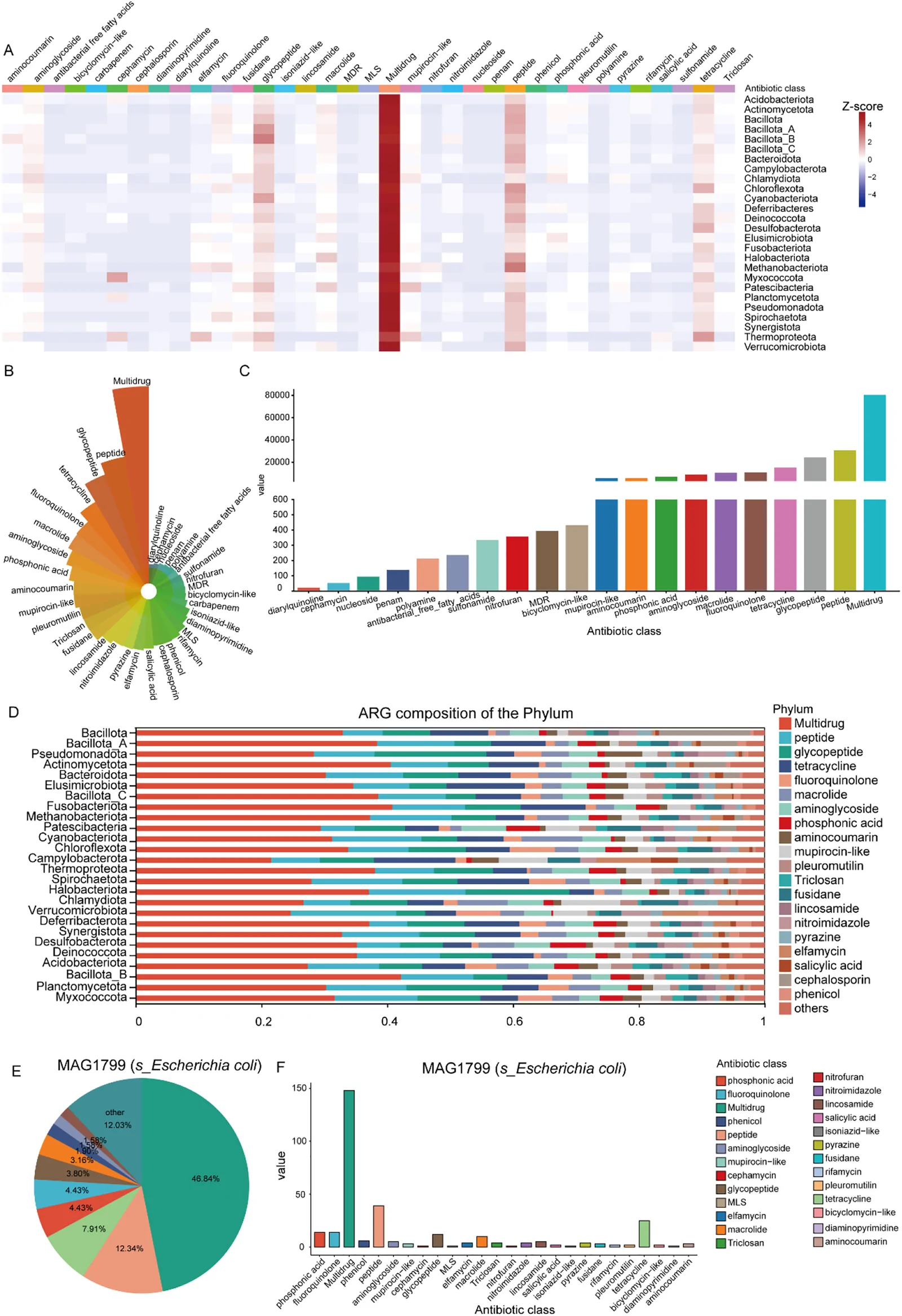
ARG profile of chicken gut MAGs. **A** Heatmap of ARG abundance across MAGs. Color gradient reflects increasing gene counts. **B** Summary of all ARG classes detected. **C** Top 10 and bottom 10 ARGs by abundance. **D** Phylum-level distribution of ARGs. E Pie chart of ARG categories in MAG1799 (*Escherichia coli*). **F** Bar chart showing the number of ARGs per resistance category in MAG1799 (*Escherichia coli*).

To characterize the antibiotic resistome, we analyzed 2,146 high-quality MAGs for ARG content using the CARD database. A total of 254,316 ARGs were identified, including 159 unique genes across 35 antibiotic resistance categories (Fig. 4A, B). All MAGs harbored at least 17 ARGs, with multidrug (81,062), peptide (31,749), glycopeptide (25,135), and tetracycline (15,963) resistance genes being most prevalent. These four categories accounted for over 65% of total ARGs, underscoring the widespread dissemination of resistance traits in the chicken gut microbiome (Table S5).

ARGs were not evenly distributed across taxa. Multidrug and tetracycline resistance genes were especially enriched in MAGs from Bacillota, Bacillota_A, Pseudomonadota, and Actinomycetota, indicating these phyla as key reservoirs. Additionally, ARGs (661 total) were detected in archaeal lineages, including methanogens and Thermoproteobacteria (Fig. 4C). Resistance genes for multidrug, peptide, and glycopeptide antibiotics were broadly distributed across MAGs (Fig. 4D), suggesting widespread potential for antimicrobial resistance transmission within the gut ecosystem.

BacMet analysis again highlighted MAG2050 (Klebsiella pneumoniae), MAG1267 (Janthinobacterium sp001758725), and MAG1655 (Leclercia adecarboxylata_C) as encoding the highest numbers of BacMet genes (429, 400, and 343, respectively). Notably, MAG1799 (E. coli) was found to harbor 326 unique ARGs spanning 27 drug resistance classes (Fig. 4E, F), including resistance to multidrug, peptide, tetracycline, glycopeptide, phosphonic acid, aminoglycoside, and fluoroquinolone antibiotics. Given the clinical significance of E. coli as a pathogen, this strain may represent a potential multidrug-resistant "superbug." While its antibiotic resistance could aid in laboratory isolation and cultivation, its presence also raises concerns regarding ARG dissemination and host microbiome disruption.

### 3.5 Mobile genetic elements

**Fig. 5:**
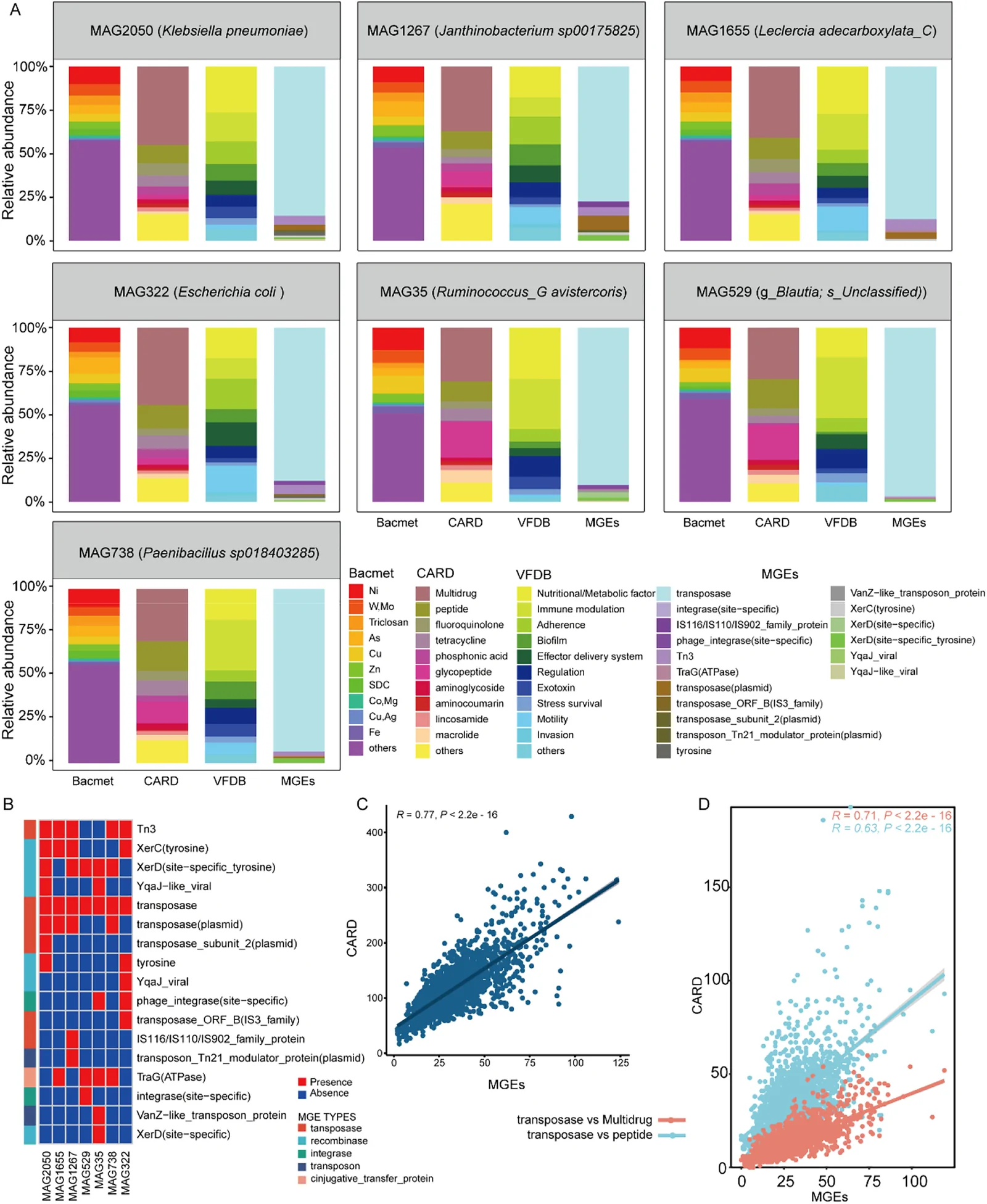
Correlation between ARGs and MGEs. **A** Bar chart showing the relative abundance of selected MAGs annotated with BacMet, CARD, VFDB, and MGEs. **B** Heatmap depicting correlations between ARG categories and MGE types. **C** Linear regression between total ARG and MGE abundance across 2,146 MAGs. **D** Correlation between multidrug/peptide ARGs and transposase abundance.

To assess the potential for horizontal gene transfer of ARGs in the chicken gut microbiome, we examined the association between ARGs and MGEs. On average, 33.84 MGEs were identified per MAG. Notably, MAG35 (Ruminococcus_G avistercoris), MAG529 (g_Blautia; s_Unclassified), and MAG738 (Paenibacillus sp018403285) contained the highest numbers of MGEs—124, 123, and 106, respectively (Table S4). At the species level, Ligilactobacillus agilis (1,781 MGEs), Limosilactobacillus ingluviei (1,227), g_Lactobacillus; s_Unclassified (1,225), Ligilactobacillus salivarius (1,020), and E. coli (785) exhibited the highest total MGE counts (Fig. 5A).

We further analyzed the relationship between MGE and ARG abundance. A correlation heatmap generated using the ggcorrplot package revealed strong associations between specific MGE types and ARG categories (Fig. 5B). A significant linear relationship was observed between overall MGE and ARG abundance (y = 0.2723x + 1.5681, R = 0.77) (Fig. 5C), suggesting that MGEs play a critical role in ARG dissemination. Specifically, multidrug and peptide resistance gene abundance showed strong positive correlations with transposase elements (R = 0.71 and R = 0.63, respectively; both *P* < 0.001) (Fig. 5D), underscoring the importance of MGEs in mediating the horizontal transfer of resistance traits.

### 3.6 Microbial diversity and differentially abundant MAGs across four provinces

**Fig. 6:**
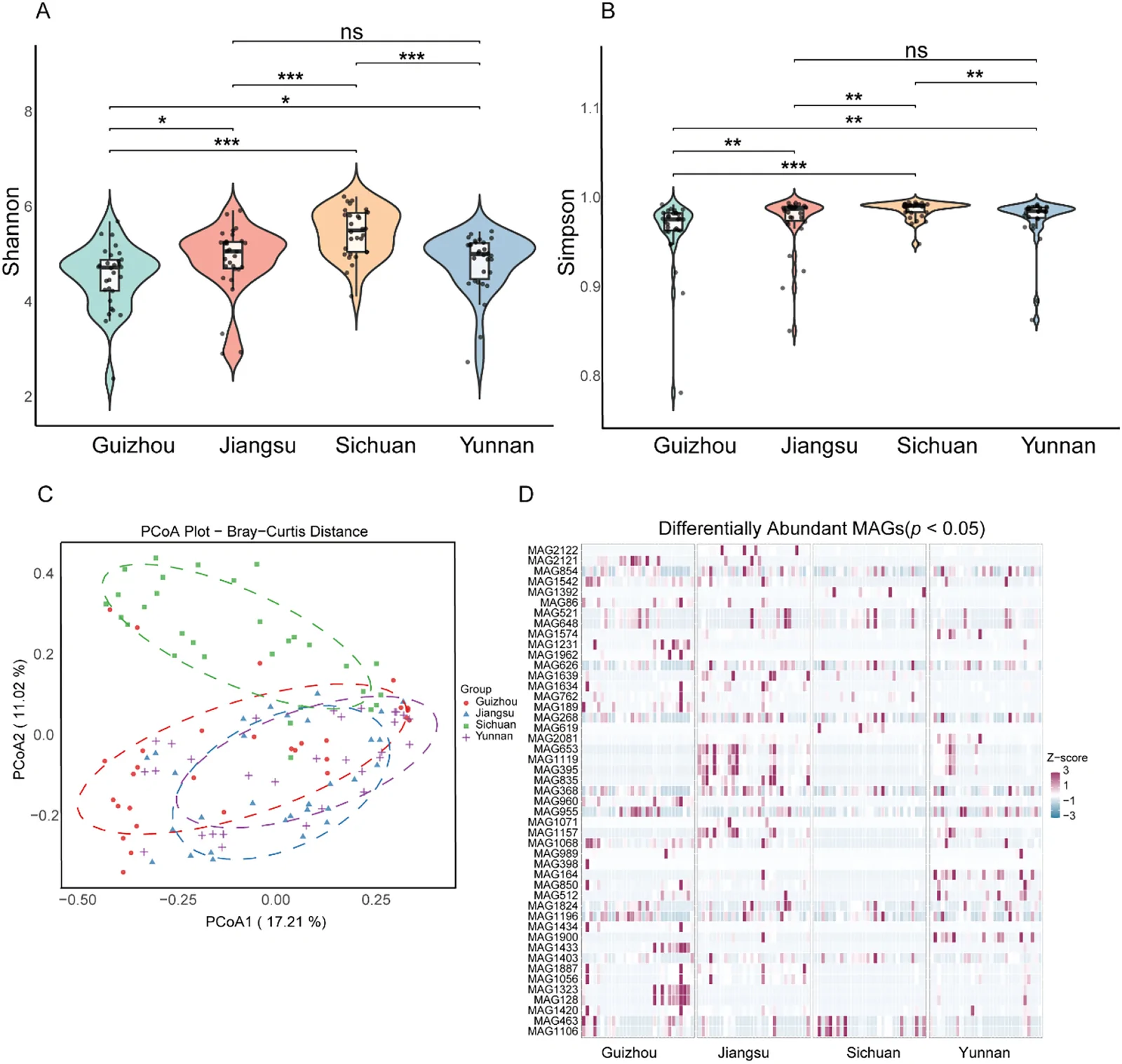
Microbial diversity and differentially abundant MAGs across four provinces. **A** Shannon diversity index comparisons across provinces. **B** Simpson diversity index comparisons. **C** PCoA plot based on Bray–Curtis distances illustrating differences in microbial community structure among the four regions. Statistical significance is indicated as *p* < 0.05 (*), *p* < 0.01 (**), *p* < 0.001 (***), and non-significant (ns). **D** Heatmap showing Z-score–normalized abundances of significantly different MAGs among four provinces.

Alpha-diversity analyses demonstrated clear regional differences in the richness and evenness of the MAG-based microbial communities. The Shannon index was significantly higher in samples from Sichuan compared with those from other three provinces (multiple comparisons, *p* < 0.05) (Fig. 6A), a similar pattern was observed for the Simpson index (Fig. 6B). Beta-diversity analysis using Bray–Curtis distances further indicated distinct community structures across provinces. PCoA revealed clear clustering by region, with Guizhou, Jiangsu, Sichuan, and Yunnan forming separated clusters along the first two principal coordinates (PCoA1 explaining 17.21% of variance; PCoA2 explaining 11.02%). This supports strong geographic structuring of microbial community composition (Fig. 6C). A total of 40 high-quality MAGs exhibited significant differential abundance among the four regions (*p* < 0.05) using ANCOM-BC modeling method. Several MAGs, such as MAG128 (*g__Atopobium*), MAG1433 (f__Atopobiaceae), and MAG1323 (f__Atopobiaceae), were enriched specifically in Guizhou samples, whereas others (e.g., MAG164 (*s__Streptococcus alactolyticus*), MAG1574 (*s__Enterococcus_B hirae*), MAG1900 (*s__Streptococcus alactolyticus*)) were more abundant in Yunnan (Fig. 6D). Taken together, these results highlight pronounced geographic differentiation in both microbial diversity and MAG-level community structure across four provinces.

### 3.7 The ARG-MGE adjacented relationship in the MAGs

**Fig. 7:**
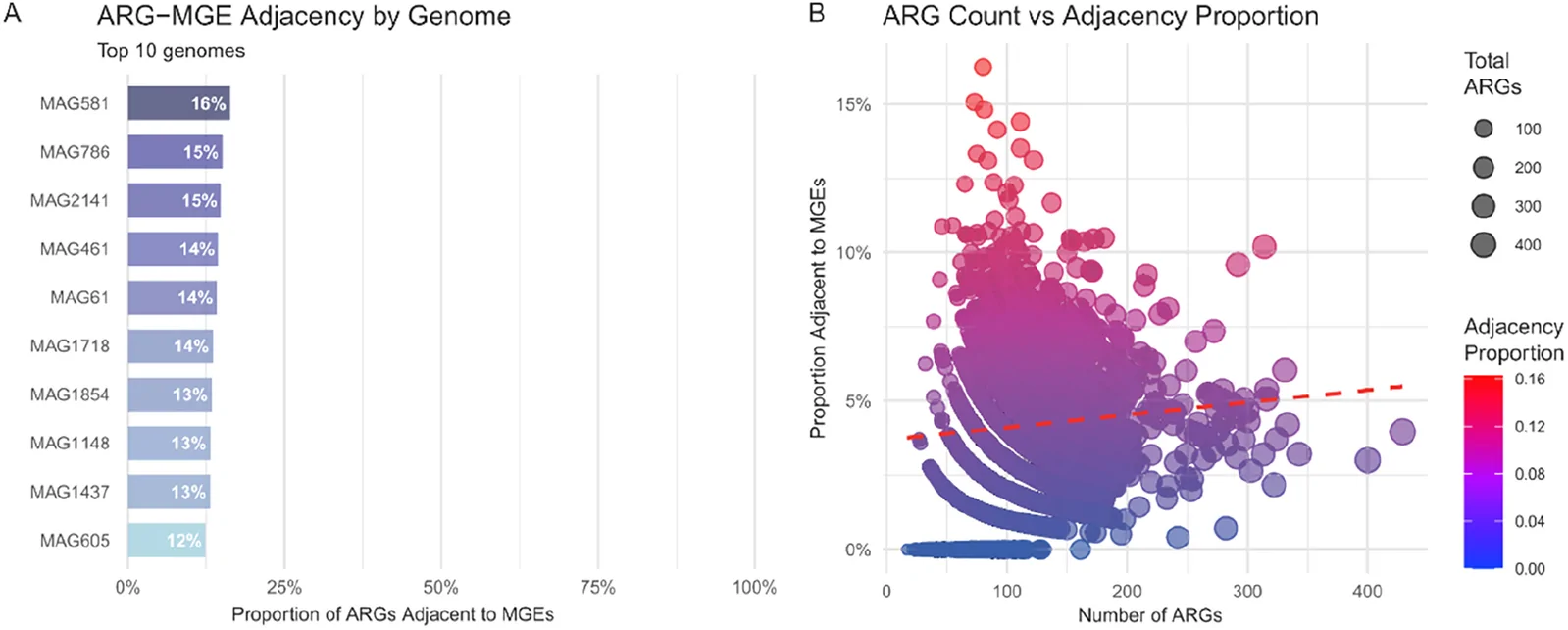
Analysis of the Adjacent relationship between ARGs and MGEs. **A** The top 10 genomes with the highest adjacented ratio of ARG-MGE at the genomic level. **B** The relationship graph between the number of ARGs and the adjacented ratio, where the size of the dots indicates the total number of ARGs, the depth of the color indicates the adjacented ratio, and the red dotted line indicates the average proximity ratio.

Based on the comprehensive analysis of all genes across 2,146 bacterial genomes, we identified 254,316 ARGs and 72,617 MGEs. The genomic proximity analysis revealed that 10,826 ARGs (4.26%) were located adjacent to MGEs, supporting the hypothesis of horizontal gene transfer facilitation, and 133 MAGs were both sides adjacent to MGEs. Among the MAGs analyzed, MAG581 (*g__Lactobacillus*, 16.25%) exhibited the highest proportion of ARG-MGE adjacency, followed by MAG786 (*s__Lactobacillus pasteurii*, 15.07%) and MAG2141 (*s__Lactobacillus johnsonii*, 14.81%) (Fig. 7CD). This non-uniform distribution of ARG-MGE proximity across different genomes suggests varying potentials for horizontal transfer of antibiotic resistance determinants within this microbial community. The frequent co-localization of ARGs with MGEs underscores the important role of mobile elements in the dissemination of antibiotic resistance.

## 4 Discussion

### 4.1 Genome-resolved metagenomics expands our understanding of the poultry gut microbial ecosystem

Genome-resolved metagenomics has increasingly been recognized as a powerful tool for studying complex microbial ecosystems, as it allows the direct association of functional genes with specific microbial taxa. This approach has revealed that animal gut microbiomes represent major reservoirs of ARGs and MGEs that can potentially contribute to the global dissemination of antimicrobial resistance (Kayani et al., 2021; Kim et al., 2024). From a microbial ecology perspective, the chicken gut represents a dynamic environment where microbial populations interact through metabolic exchange, competition, and horizontal gene transfer, shaping the structure and evolution of the resistome.

In this study, 2,146 MAGs were reconstructed from 120 fecal samples of free-range chickens from four provinces to analyze the ARGs distribution in four provinces in China. This number is greater than previous studies (such as only 155 and 409 draft microbial genomes were reconstructed from chicken samples)(Segura-Wang et al., 2021; Xu et al., 2022); another study used high-fidelity long reads from the 5 chicken intestinal compartments and assembled 461 and 337 microbial genomes (Zhang et al., 2022). A recent CMKMC study aimed to construct a comprehensive multi-kingdom genomic catalog of the chicken gastrointestinal microbiome, including bacteria, archaea, and viruses (Y. Wang et al., 2024). In contrast, our study focuses specifically on genome-resolved resistome characterization in free-range chickens across multiple Chinese provinces, linking ARGs and MGEs to specific microbial genomes. In addition, we used short-read shotgun metagenomics and genome-resolved binning to profile the chicken gut resistome and to assign ARGs to MAGs, another study (Xiao et al., 2023) did not annotate these ARGs to MAGs. Recent studies have extensively investigated the chicken gut resistome in China using metagenomic sequencing, revealing substantial diversity and widespread distribution of ARGs in poultry systems (Segura-Wang et al., 2021; Wang et al., 2021; Y. Wang et al., 2024; Xiao et al., 2023; Xu et al., 2022; J. Yang et al., 2022). Most of these studies employed read-based or gene-catalog metagenomic approaches, which provide high sensitivity for ARG detection but have limited ability to assign ARGs to specific microbial hosts. In contrast, the genome-resolved metagenomic strategy used in this study reconstructs MAGs, enabling more precise host attribution and genomic context analysis of ARGs.

### 4.2 The poultry gut microbiome harbors a diverse and functionally redundant resistome

ARGs are widely distributed across environmental matrices, including soil, water, air, and host-associated microbiomes (Davies & Davies, 2010; Tong et al., 2024; Zhuang et al., 2021), posing serious risks to both ecosystem stability and public health (X. Wang et al., 2024). Here, we identified 254,316 ARGs representing 159 resistance gene types across 35 antibiotic classes, highlighting the remarkable diversity of resistance determinants present in the chicken gut microbiome. Among these, multidrug, tetracycline, glycopeptide, and peptide resistance genes were the most abundant categories. Similar resistance profiles have been reported in livestock microbiomes worldwide, where tetracycline resistance genes are consistently dominant due to their historical and widespread use in animal husbandry (Lawther et al., 2022; Pal et al., 2016).

From an ecological perspective, the dominance of multidrug resistance genes may reflect strong selective pressures imposed by antimicrobial exposure and other environmental stressors. Many multidrug resistance genes encode efflux pumps, which provide broad-spectrum resistance and enhance bacterial adaptability under fluctuating environmental conditions (Huang et al., 2022). Moreover, the persistence of tetracycline resistance genes even in antibiotic-free systems suggests that these genes may be maintained through co-selection mechanisms, such as linkage with heavy metal resistance genes or MGEs (Kang et al., 2018). Interestingly, ARGs were detected not only in bacterial MAGs but also in archaeal genomes. Although archaea have traditionally been overlooked in resistome studies, emerging evidence suggests that archaeal communities may participate in microbial gene exchange networks within complex microbiomes (Hou et al., 2025). This finding highlights the importance of considering the entire microbial community when evaluating antimicrobial resistance reservoirs.

In addition to microbial interactions, host-associated and environmental factors may also shape the resistome structure. Regional comparisons further revealed that chickens in China harbor a higher abundance of ARGs but lower gene diversity compared with European chickens (Feng et al., 2023). This contrast underscores the profound influence of regional farming practices and environmental conditions on gut resistome composition. However, when comparing across host species, including humans, host-specific factors outweigh regional influences in shaping ARG profiles, highlighting the dominant role of host-associated selective pressures in microbial resistance evolution (Ruppé et al., 2019). The observed diversity and abundance of ARGs in chicken gut samples likely reflect ecological niches shaped by host physiology, diet, and antibiotic exposure (Liu et al., 2020; Wang et al., 2024, 2023). Consistent with these ecological dynamics, tetracycline and aminoglycoside resistance genes were particularly prevalent in several chicken gut samples. Comparable resistance patterns have also been observed in other livestock-associated microbiomes. For instance, in pig respiratory tract samples, resistance genes for tetracyclines and aminoglycosides accounted for approximately 16% and 14%, respectively (Ye et al., 2025). Together, these observations suggest that prolonged antibiotic use in livestock production has exerted substantial selective pressure, facilitating the evolution and maintenance of resistance mechanisms among gut microbes (Li et al., 2024).

Additionally, multi-omics approaches including genomics, metagenomics, and transcriptomics have become essential for elucidating animal traits and adaptive mechanisms (T. Wang, P. Li, et al., 2024; Wang et al., 2025). Recent advances in single-cell transcriptomics, for instance, have provided deep insights into avian performance and physiological adaptation (Leng et al., 2024; T. Wang, D. Leng, et al., 2024), underscoring the power of integrative omics frameworks in agricultural and ecological research.

### 4.3 Mobile genetic elements drive resistome plasticity in the chicken gut microbiome

As key vectors for ARG transmission, MGEs are closely intertwined with resistance gene dissemination (Forster et al., 2022; Wu et al., 2022). MGEs such as plasmids, transposons, insertion sequences, and integrons are key facilitators of horizontal gene transfer, often carrying multiple ARGs and enabling their rapid dissemination across bacterial populations (Botelho et al., 2019). In our study, we identified 72,617 MGEs, with an average of 33.8 MGEs per MAG, and observed a strong positive correlation between ARG abundance and MGE abundance (*R* = 0.77). These results suggest that horizontal gene transfer plays a critical role in shaping the resistome structure within the chicken gut ecosystem. Unlike previous studies that relied on short-read-based taxonomic profiling, our MAG-based approach enables linkage of ARGs to specific bacterial lineages and mobile elements, offering a more accurate assessment of horizontal transfer potential. A previous analysis of 152 gene exchange networks involving 22,963 bacterial genomes identified numerous mobile element genes surrounding ARGs, reinforcing the central role of MGEs in mediating horizontal gene transfer (Ellabaan et al., 2021). Further evidence from studies on small-scale pig farms and adjacent environments has demonstrated that MGEs facilitate ARG transfer across multiple media, including manure, soil, ditch sediment, and tidal flats, with *tnpA* and *IS26* identified as major hubs of connectivity (Yu et al., 2024). These findings demonstrate that MGEs not only facilitate intra-genomic ARG movement but also drive cross-environmental spread, thereby amplifying the ecological risk posed by antimicrobial resistance.

Our genomic proximity analysis further revealed that 10,826 ARGs (4.26%) were located adjacent to MGE-associated genes, indicating a substantial pool of potentially mobilizable resistance determinants. The co-localization of ARGs with transposases, integrases, and insertion sequence elements is widely considered a hallmark of mobile resistance units and has been reported across diverse microbiomes (Zhang et al., 2025). For example, large-scale genomic analyses have shown that ARG–MGE associations form complex gene exchange networks that facilitate the rapid dissemination of resistance genes across bacterial lineages (Ellabaan et al., 2021). Recent studies have also highlighted the role of specific mobile elements, such as IS26-associated transposons and plasmid-borne integrons, in promoting the spread of clinically relevant resistance genes across animal and environmental microbiomes (Harmer & Hall, 2024; Harmer et al., 2020). The widespread ARG–MGE associations observed in our study therefore indicate that the poultry gut microbiome may function as an active hotspot for horizontal gene transfer.

### 4.4 Geographic heterogeneity reflects environmental and management influences on poultry resistomes

Our results revealed clear geographic variation in both microbial community composition and ARG distribution among the four provinces studied. Such regional differences are consistent with previous reports showing that livestock gut microbiomes are strongly shaped by diet, farm management practices, environmental microbial exposure, and local ecological conditions (Han et al., 2025; Williams et al., 2023; Zhou et al., 2025).

Environmental reservoirs surrounding farms, including soil, water, and manure, can contribute to the circulation of ARGs between animals and the environment, forming interconnected resistome networks (Bai et al., 2025). Differences in climate, agricultural practices, and antibiotic usage policies across regions may therefore influence the selective pressures acting on microbial communities, ultimately shaping the composition of the gut resistome. These findings highlight the importance of considering regional ecological contexts when assessing antimicrobial resistance risks in livestock production systems.

### 4.5 One Health implications: poultry microbiomes as reservoirs of transmissible resistance genes

Antimicrobial resistance is increasingly recognized as a One Health challenge, linking human, animal, and environmental health systems. Livestock production systems, particularly intensive poultry farming, represent major reservoirs of resistance genes that may disseminate through food chains, agricultural runoff, and environmental contamination. Recent studies have demonstrated that ARGs originating from animal microbiomes can be detected in surrounding environmental compartments and even in human-associated microbiomes, suggesting potential transmission routes across ecological boundaries (Bai et al., 2025; Han et al., 2025). The widespread ARGs and MGEs identified in our study therefore underscore the potential role of poultry microbiomes in the broader ecology of antimicrobial resistance.

From a surveillance perspective, genome-resolved metagenomics offers a powerful framework for identifying high-risk microbial hosts and mobile ARG cassettes that may contribute disproportionately to resistance dissemination. Integrating such approaches into One Health monitoring programs could substantially improve our ability to track resistance gene flow across animal, environmental, and human microbiomes. Future studies should therefore focus on longitudinal monitoring and cross-ecosystem comparisons to better understand how poultry-associated resistomes interact with environmental and human microbial communities.

## 5 Conclusion

Metagenomic profiling of the chicken gut microbiota reveals a complex and dynamic network of microbial taxa and antibiotic resistance genes, shaped by both host and environmental factors. These results emphasize the need to integrate ecological, genomic, and epidemiological data when devising strategies to mitigate antibiotic resistance. Future investigations should focus on functionally characterizing resistance determinants, elucidating the mechanisms of ARG–MGE interactions, and implementing evidence-based interventions to reduce the transmission of resistance in animal agriculture.

## Ethical approval

The collection of fecal samples used in this study was reviewed and approved by the Ethics Committee of Chengdu University (Approval No. 20220716).

## CRediT authorship contribution statement

**Dongming Qi:** Writing – review & editing, Writing – original draft, Conceptualization. **Jinyuan Liu:** Writing – review & editing, Writing – original draft, Formal analysis. **Xue Bai:** Writing – review & editing, Formal analysis. **Binlong Chen:** Writing – review & editing, Conceptualization. **Fengjiao Qiu:** Writing – review & editing, Formal analysis. **Caiyun Sun:** Writing – review & editing, Data curation. **Jianyong An:** Writing – review & editing, Formal analysis. **Anqiang Lai:** Writing – review & editing, Formal analysis. **Xiaoyan Li:** Writing – review & editing, Conceptualization.

## Declaration of competing interest

The authors declare that they have no known competing financial interests or personal relationships that could have appeared to influence the work reported in this paper.

## Data Availability

The original data presented in this study are openly available in the NCBI (https://www.ncbi.nlm.nih.gov/) under Bioproject accession number PRJNA1039992. The codes are available at https://github.com/JinyuanLiu123/Chicken-MAG/tree/master.
